# Regulatory network characterization in development: challenges and opportunities

**DOI:** 10.12688/f1000research.15271.1

**Published:** 2018-09-17

**Authors:** Guangdun Peng, Jing-Dong J. Han

**Affiliations:** 1 CAS Key Laboratory of Regenerative Biology and Guangdong Provincial Key Laboratory of Stem Cell and Regenerative Medicine, Guangzhou Institutes of Biomedicine and Health, Chinese Academy of Sciences, Guangzhou, China; 2 Guangzhou Regenerative Medicine and Health Guangdong Laboratory, Guangzhou, China; 3 Key Laboratory of Computational Biology, CAS Center for Excellence in Molecular Cell Science, Collaborative Innovation Center for Genetics and Developmental Biology, Chinese Academy of Sciences-Max Planck Partner Institute for Computational Biology, Shanghai Institutes for Biological Sciences, Chinese Academy of Sciences, Shanghai, China

**Keywords:** gene regulatory network, single-cell RNA-seq, development, Transcription factor, spatial transcriptome

## Abstract

Embryonic development and stem cell differentiation, during which coordinated cell fate specification takes place in a spatial and temporal context, serve as a paradigm for studying the orderly assembly of gene regulatory networks (GRNs) and the fundamental mechanism of GRNs in driving lineage determination. However, knowledge of reliable GRN annotation for dynamic development regulation, particularly for unveiling the complex temporal and spatial architecture of tissue stem cells, remains inadequate. With the advent of single-cell RNA sequencing technology, elucidating GRNs in development and stem cell processes poses both new challenges and unprecedented opportunities. This review takes a snapshot of some of this work and its implication in the regulative nature of early mammalian development and specification of the distinct cell types during embryogenesis.

## Introduction

In recent years, stem cells and stem cell-based translational applications have been recognized as a promising strategy in the future of medicine to tackle incurable situations by conventional treatment (for example, neural degenerative diseases and organ failure). However, one of the major obstacles for stem cell therapy is the low purity with low efficiency in obtaining functional cells because of the lack of a complete understanding of *in vivo* stem cell lineage (that is, the normal developmental processes), which generates the authentic and functional cell types with high efficiency.

Embryonic early development is tightly controlled by intrinsic and extrinsic factors. The activity of the transcription factors (TFs), microRNAs, and related gene regulatory networks (GRNs)—as significant intrinsic regulators—is essential for the maintenance of pluripotent states and orchestrated specification of progenitor fates. However, despite accumulated studies in molecular, cellular, and animal levels that have profoundly revealed the key players during early development, the dynamic interaction of GRNs—with their large number of components and even larger number of potential interactions between those components—demands a systematic and high-dimensional approach. Moreover, building detailed predictive computational models of GRNs based on the high-dimensional data is challenging.

In this article, we briefly review the regulation of early development and focus on recent advances of enabling technologies and methodologies—for example, single-cell RNA sequencing (scRNA-seq) and spatial transcriptome—in characterizing the GRNs of early embryo development.

## Cell fate determination and lineage specification of early embryo development

Early embryo development in vertebrate animals is conserved in molecular regulations ^1^. In mouse embryo development, for example, the zygote cell undergoes sequential cell divisions and two major cell fate segregations before proceeding to germ layer determination. The first lineage segregation occurs shortly after fertilization, during which the totipotent blastomeres give rise to the inner cell mass (ICM) and the trophectoderm. ICM cells are a pluripotent cell population from which all cell types in the embryo proper, as well as tissues of the extraembryonic fetal membranes, will be generated, while the trophectoderm will contribute to tissues of the fetal components of placenta. The ICM gives rise to the epiblast and the primitive endoderm at the second lineage segregation. Afterwards, the embryo goes through a continuum of pluripotent states such as the continuous transition from naïve, formative to primed pluripotency ^2^ and forms the primary germ layers that eventually set the body plan ^3^.

The remarkable similarity in the stem cell behavior of animal species during periods of early embryonic development points to the existence of an inherent conserved molecular principle underpinning the cell fate determination ^4, 5^. It is now known that during this complex process, stem cell hierarchical systems are established with step-wise restricted differentiating capacities following the orchestration of transcriptional regulation, through which the encoding and coordinating morphogenetic outcomes are attained ^1, 6^. Moreover, there exist intricate causal relationships between the cell type-specific GRNs and the phenotypic outputs during embryo development and stem cell differentiation, making the understanding of gene regulation a demanding task.

## Systematic approaches to study transcription regulation for the development process

The particular architecture and dynamics of cell type-specific GRNs that contribute profoundly to tissue organization during development have been conventionally studied by a gene-by-gene approach (for example, genetic manipulation and lineage tracing). A compendium of TFs and molecular determinants that are involved in pluripotency maintenance and cell fate determination has been extensively described (summarized in 7, 8). Though limited by the inherent incompleteness of low-throughput methods, these factors have been cornerstones for high-throughput and systematic studies to build reliable networks and to verify computational modeling and simulation.

Molecular characterization of cell identity and the annotation of the GRNs using next-generation sequencing technologies have opened up new avenues to dissect the developmental events and reconstruct the cell lineage in unprecedented detail. The high volume of data enables the possibilities of understanding gene regulation for cell programming and reprogramming in an unbiased manner, which in many cases greatly facilitates the discovery of new findings and novel players ^3^. For example, the state of stem cell pluripotency is stabilized by an interconnected pluripotency gene network consisting of TFs, TF downstream targets, and microRNAs ^9– 11^. Stem cells integrate external signals and internal molecular programs to exert control over the decision between self-renewal and differentiation. The GRNs in this context have profound implications for differentiation and trans-differentiation ^10^. Accordingly, a systematic integration of the network biology platform named CellNet enables directed and enhanced cell fate conversion by reconstruction of cell type-specific GRNs and regulatory nodes that determine whether engineered cells are equivalent to their target tissues ^12, 13^.

Compared with embryo development of a few cells in the first two cell fate decisions, there is combinatorial activity of GRNs that is deployed in the temporal and spatial context to ensure the transition and exit of the multipotent epiblast from pluripotency to lineage differentiation at gastrulation stages ^14– 17^. Genome-wide transcription activity underlining gastrulation and organogenesis has been profiled and the results indicated that there are distinctive and coordinated switches in the gene expression patterns ^18^. However, as many GRNs are partitioned and regionalized in a spatially ordered manner to proclaim the cell fates, it is vital that the GRN profiling be revealed in the dynamic embryonic positions ^19^. To this end, a spatially resolved transcriptomic analysis based on laser microdissection in the mid-gastrulation mouse embryo pinpointing the discrete transcriptomic profiles and signaling network that establish the anterior–posterior patterning has been reported ^20^. This analysis provided a proof-of-concept model that GRN organization in real space can be interrogated and correlated to diversified cell fates, which enhances the understanding of development regulation in native environmental settings, as happens *in vivo*.

Recently, accumulating evidence suggested that epigenetic modifications on chromosome structure and accessibility are highly relevant for the establishment of the GRN ^21, 22^. Development-related TF genes can be modulated by chromatin states, and an intricate interaction between these two has been shown to be essential for proper stem cell differentiation and germ layer specification ^15^. A combination analysis of GRN, epigenome, and signaling network thus constitutes the quantitative understanding of the development process.

## Single-cell approaches to study transcription regulation for the development process

Developmental events do not take place abruptly. In most cases, complex mixtures of molecular behaviors are tightly coupled and happen sequentially during cell fate determination and pluripotency exit, making conventional GRN analysis based on physically separated cell populations very challenging. The strictly controlled and orderly changes in cell identity transition presume that there is a smooth and continuous cell status embedded in single cells. Therefore, the cutting-edge single-cell technologies, which now can assay RNA, DNA, DNA methylation, histone modifications, and chromosome accessibility of thousands of single cells simultaneously, have become a revolutionary tool to capture continuous changes and decipher GRNs in the developmental process ^23^.

Driven by and reflected in molecular changes of GRNs, cells adopt their distinct fates following an asynchronous branching pathway of development as depicted in Waddington’s landscape ^24^. If enough cells are analyzed, the transition paths to the terminally differentiated cell types of single cells (that is, developmental trajectory) can be reconstructed by calculating transcriptomic similarity and distances. Therefore, it is vital that the proper sequencing strategy with consideration of sampling size, tissue complexity, and sequencing depth has been employed to precisely define the developmental tree. For example, greater sequencing depths and more captured single cells are required for regulatory network analysis of the developmental process with complex branches.

With a large enough number of single cells representing potentially omnipresent states, pseudotemporal ordering algorithms have been developed to place cells along the developmental trajectory to reveal the lineage relationship that is encoded in the gene expression similarity. Various computational tools based on this assumption have been developed to model the developmental process and single-cell behaviors in scRNA-seq data ^25^. For example, Monocle reduces the data dimensionality into essential ones and takes advantage of the minimum spanning tree to calculate the developmental path ^26^. Diffusion pseudotime based on diffusion-like random walk distances was applied to map developmental branching decisions ^27^. Importantly, as spatial information significantly contributes to the cellular states, pseudospace can be potentially uncovered by using a similar approach ^28^. However, these pseudotime methodologies encounter difficulties in accurately reconstructing branching trajectories in the event that more than one path derives from a single point or from multiple origins, as often happens in *in vivo* development (as shown in recent studies ^29, 30^). The main assumption and an intrinsic limitation of the pseudotime reconstruction is that the gene expression similarity reveals the lineage relationship, which sometimes is not real, as there are discontinuous cell states, such as asymmetrical cell division ^31^, not to mention that many transcriptome similarities, such as common cell cycle or metabolic states ^32, 33^, are irrelevant to lineage relationships and that factors other than the transcriptome, such as metabolism regulation and splicing regulation, are also vital for lineage differentiation ^34– 37^. Moreover, the performance and robustness of these pseudotime methods are difficult to benchmark because of large diversity in the outputted data structures and the lack of authentic experimental replicates. In addition, confounding factors, such as cell cycle phases, must be excluded in such single-cell transcriptome similarity-based trajectory reconstruction ^32^. This, however, precludes the study of the role of the cell cycle in differentiation and development. To circumvent such a limitation, Sun *et al*. developed a method to reconstruct the single-cell developmental trajectory by using matching cell population data as an external reference; using such an approach revealed that the M-phase exit check point and its regulation control neural differentiation speed at the single-cell level ^33^. Another advantage of the cell population reference-based trajectory inferences is that the time of differentiation is no longer a pseudotime but a predicted time scaled to and benchmarked by the real different time ^33^.

Coupling information from different expression modalities along developmental trajectories with transcriptional regulation possibly enables the delineation of cell hierarchy and rare intermediate cell states and unveils the regulatory networks in many details. To this end, a variety of computational methods for inferring GRNs with single-cell data have been rigorously tested. For instance, to reveal the regulatory network from single-cell data, SCENIC (single-cell regulatory network inference and clustering) defines the cell states via binarization of the single-cell data and links the co-expression modules with cis-regulatory sequences. The regulon activity was constructed and scored in each cell. The regulatory network based on scored regulon facilitates a mechanistic interpretation of the data because of the inclusion of motif information ^38^. This method has been exploited to guide the identification of TFs and cell states ^39^. Boolean simulation models randomly pick genes and toggle them asynchronously to predict the cell fate transition and heterogonous cell response, which has been used to recapitulate signal-dependent cell differentiation ^40^. Weighted gene co-expression network analysis (WGCNA) was also used to construct the regulatory network, to determine functional key players of embryonic development, and to uncover potential functional modules ^41, 42^. Connection specificity index analysis takes inputs of the gene co-expression matrix to identify significant interaction-profile similarities and define modules of genes with similar profiles ^43^, where gene pairs with a cutoff above statistically significant relationships result in a relevance network shown in the graph matrix. The edges in the matrix between genes denote potential interactions. The positive edges defined by a positive correlation indicate a potential activation, and negative edges indicate a potential inhibitory relationship ^44^. With this analysis, a spatially interacted TF network has been shown in establishing the anterior and posterior patterning during mouse gastrulation ^20^, and TFs in maintaining single-cell lineages were also collated ^45^.

## The challenges for gene regulatory network analysis for single cells

**Figure 1.**
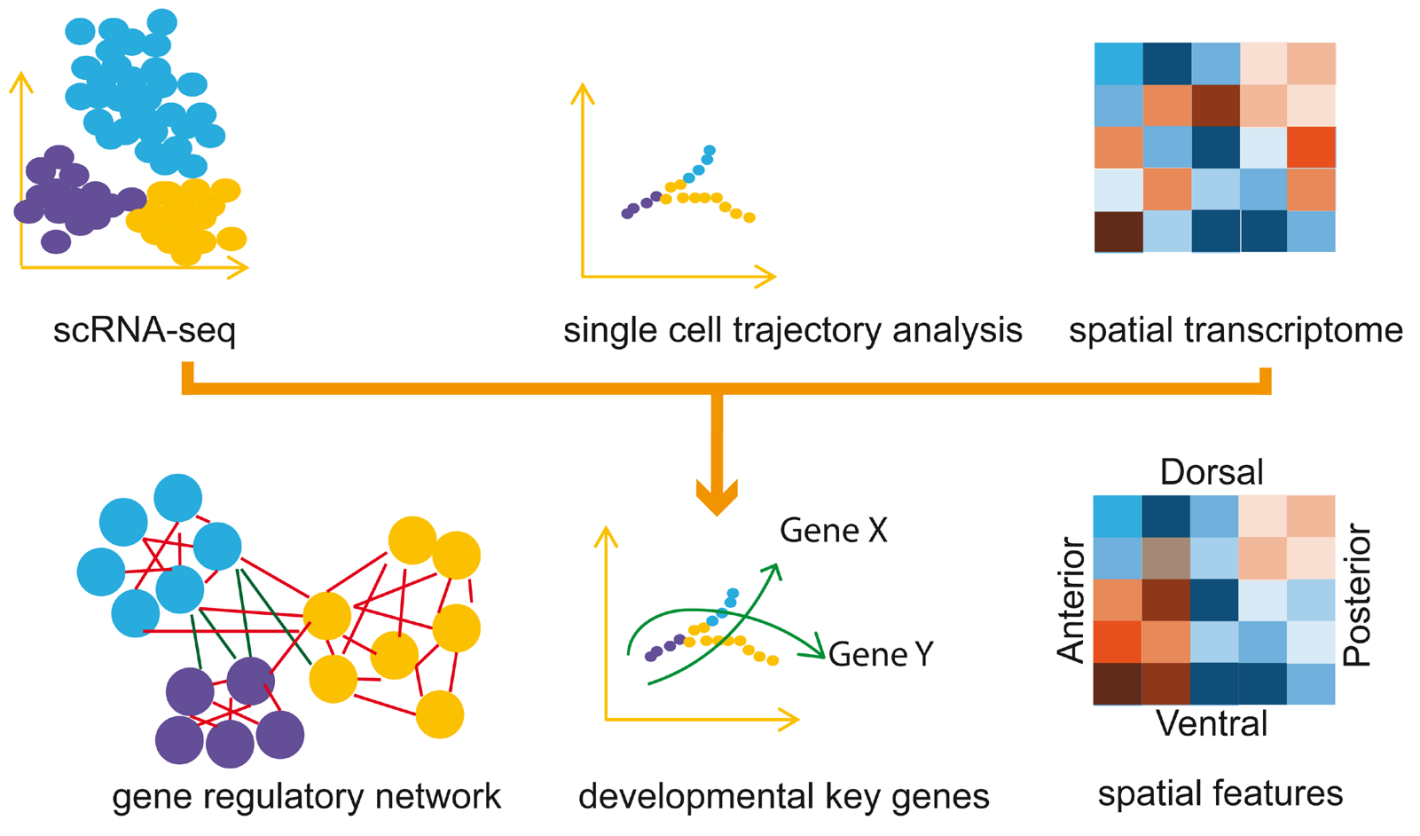
Gene regulatory network inference from single-cell and spatial transcriptome data. Single-cell RNA sequencing (scRNA-seq) data are subjected to dimension reduction and path finding to reconstruct the trajectory. Combined with spatial transcriptome data, the gene regulatory networks can be inferred (see ‘The challenges for gene regulatory network analysis for single cells’ section details) to provide explanations for the developmental process and spatial patterning.

GRNs are essentially dictated by the genetic hierarchy of the TF network, cis-regulatory elements, and downstream targets. In this regard, critical experimental validation by various perturbations should be combined to test the computational model. However, GRNs inferred from single-cell data are prone to be difficult in cross-validation because of the inherent stochastic nature of single cells. First, individual cells have a variable mRNA content that is unpredictable, and transcription activity in single cells fluctuates frequently; both contribute to the complexity and noise of scRNA-seq data ^46^. Second, the amplification of minimal RNA molecules also introduces technical noise and batch effects. Although there are improved methods to tackle the problems by using unique molecular identifiers or a 3′ targeting sequencing strategy ^47^, it remains difficult to distinguish technical noise from genuine biological variability that contains valuable information. Third, the constitution and quantitative impact of different sources of noise have not been systematically evaluated. These kinds of confounding factors can profoundly alter the structure of GRNs. Finally, the GRN in the native setting (that is, with the inputs from spatial and environmental interactions) has not been explored effectively ( Figure 1). Available methods rely on known spatial landmarks or computationally simulated spatial features to trace single cells to their spatial origins ^48– 51^. It is important that *de novo* identification of spatial coordinates and location mapping based on an unbiased method shall be established. In this regard, Peng *et al*. applied laser microdissection to systematically measure the gene expression profiles in the real location of mouse embryos and provided a zip-code utility to map single cells to their original position in the embryo ^20^. In the future, with more computational models developed, a framework to infer GRNs and their dynamics in driving the sequential cell fate determination during development from scRNA-seq data with statistical accuracy can be expected ^52^. Toward this end, Sun *et al*. have adapted a network flow optimization method to infer the regulatory events at each cellular state transition point ^33^. More systematic and unsupervised methods are expected to deliver a more global view of the GRNs driving the spatial and dynamic process of cell fate determination.

## Outlook

The *in vivo* embryo uses regulation and canalization at multiple layers to safeguard the developmental process. The multiscale integration of modular networks of gene expression and signaling, and their interaction in spatial and temporal contexts orchestrated with epigenetic cues, constitute the core for developmental mechanisms. Ideally, a comprehensive catalog of GRNs and robust computational models would be built from ChIP-seq for all cell types and all TFs under various tissue-specific or developmental processes. However, generating such data is time-consuming and often impractical. As genome-wide expression profiling is now a routine tool in experimental design and a significant amount of biological perturbation data are also available ^53^, it would be necessary for the GRNs to be computed by including as many parameters as possible and even with the help of next-generation machine learning ^54^. Recently, high-resolution epigenetic analytical tools such as single ATAC-seq (assay for transposase-accessible chromatin using sequencing) and ChIP-seq are becoming a reality ^55– 58^, and the regulatory mechanism can be revealed with the help of multiple-layer omics inputs. With all of these data integrated, the GRN in development will be inferred more accurately and will be more biologically relevant ^59^.

## Abbreviations

ChIP-seq, chromatin immunoprecipitation followed by sequencing; GRN, gene regulatory network; ICM, inner cell mass; scRNA-seq, single-cell RNA sequencing; TF, transcription factor
